# A scoping review of eye tracking metrics used to assess visuomotor behaviours of upper limb prosthesis users

**DOI:** 10.1186/s12984-023-01180-1

**Published:** 2023-04-24

**Authors:** Kodi Y. Cheng, Mayank Rehani, Jacqueline S. Hebert

**Affiliations:** 1 Division of Physical Medicine and Rehabilitation, Department of Medicine, Faculty of Medicine and Dentistry, College of Health Science, University of Alberta, Edmonton, AB Canada; 2 Department of Biomedical Engineering, Faculty of Medicine and Dentistry, College of Health Science, University of Alberta, Edmonton, AB Canada; 3 Glenrose Rehabilitation Hospital, Alberta Health Services, Edmonton, AB Canada

**Keywords:** Eye tracking, Eye metrics, Visuomotor behaviour, Visual attention, Upper limb amputation, Myoelectric prosthesis, Body-powered prosthesis, Simulated prosthesis

## Abstract

Advanced upper limb prostheses aim to restore coordinated hand and arm function. However, this objective can be difficult to quantify as coordinated movements require an intact visuomotor system. Eye tracking has recently been applied to study the visuomotor behaviours of upper limb prosthesis users by enabling the calculation of eye movement metrics. This scoping review aims to characterize the visuomotor behaviours of upper limb prosthesis users as described by eye tracking metrics, to summarize the eye tracking metrics used to describe prosthetic behaviour, and to identify gaps in the literature and potential areas for future research. A review of the literature was performed to identify articles that reported eye tracking metrics to evaluate the visual behaviours of individuals using an upper limb prosthesis. Data on the level of amputation, type of prosthetic device, type of eye tracker, primary eye metrics, secondary outcome metrics, experimental task, aims, and key findings were extracted. Seventeen studies were included in this scoping review. A consistently reported finding is that prosthesis users have a characteristic visuomotor behaviour that differs from that of individuals with intact arm function. Visual attention has been reported to be directed more towards the hand and less towards the target during object manipulation tasks. A gaze switching strategy and delay to disengage gaze from the current target has also been reported. Differences in the type of prosthetic device and experimental task have revealed some distinct gaze behaviours. Control factors have been shown to be related to gaze behaviour, while sensory feedback and training interventions have been demonstrated to reduce the visual attention associated with prosthesis use. Eye tracking metrics have also been used to assess the cognitive load and sense of agency of prosthesis users. Overall, there is evidence that eye tracking is an effective tool to quantitatively assess the visuomotor behaviour of prosthesis users and the recorded eye metrics are sensitive to change in response to various factors. Additional studies are needed to validate the eye metrics used to assess cognitive load and sense of agency in upper limb prosthesis users.

## Introduction

The goal of advanced upper limb prostheses is to restore the highly dexterous and complex capacities of the human hand. Vision is among one of the most important senses in controlling the hand during object interaction [1]. Both individuals with an anatomical hand [2] and prosthetic hand [3] use vison to preplan movements by gathering information about the external environment. Visual fixations are directed at areas of interest prior to generating a motor command. As such, visuomotor coordination is required to achieve coordinated hand and arm function. Prosthesis users have an additional requirement to visually monitor the prosthesis, given the lack of feedback sensations that are typically provided by the anatomical hand. Novel prosthetic interventions are developed to facilitate increased functionality, while also minimizing the attentional demand associated with operating these devices. However, attentional demand can be difficult to quantify. When the gaze is focussed on a target of interest, information is processed through the fovea with high visual acuity [4]. Generally, the direction of gaze corresponds with the location of overt attention, however, does not consider covert attention that is processed through the peripheral vision [4]. Nevertheless, this principle has enabled researchers to use eye movement behaviours to measure the allocation of overt visual attention and provide insights into movement planning. Indeed, it wasn’t until recently that eye tracking research has become popular as a diagnostic tool aimed at measuring visual attention [5].

Eye tracking is a technology used to record eye movements to provide objective and unbiased insights into human gaze behaviour [6]. Modern video-based eye trackers use digital cameras to capture a series of images of the eyes. Different approaches have been employed to detect the pupil location in order to calculate the point at which the eyes are fixated [7]. By quantifying the timing and location of visual fixations, the coordination between eye and hand movements can be studied under different conditions to reveal important aspects of object interaction [2]. With the anatomical limb, the eyes precede the actions of the hands to provide movement planning information to successfully reach and grasp for target objects [8–11]. Eye tracking has been applied to identify biomarkers for cognitive impairment, as well as to track treatment progress in clinical populations such as autism spectrum disorder, schizophrenia, attention deficit hyperactivity disorder, and fetal alcohol spectrum disorder [12]. More recently, eye tracking has been utilized to evaluate the visuomotor behaviours of upper limb prosthesis users. Research in this area has further contributed to our understanding of human–machine interaction with prosthetic devices and provides a new way of potentially quantifying the usability of these devices. Currently, there is an absence of any review of eye tracking metrics within the upper limb prosthetic population. Therefore, with this growing body of literature surrounding the visuomotor behaviours of prosthesis users, there is an emergent need for a review of the literature.

A scoping review was designed to answer the question: what is known about the visuomotor behaviour of upper limb prosthesis users and which eye metrics have been used to evaluate prosthesis use? The aim of this scoping review was to identify the literature on the use of eye tracking to evaluate the behaviour of individuals using an upper limb prosthesis. In doing so, visual behaviours of prosthesis users were summarized, as well as the eye metrics used to describe these behaviours. Additionally, the literature search uncovered novel eye metrics beyond eye-hand coordination that show promise in assessing other features of prosthetic behaviour. This review paper serves to provide an understanding of how eye tracking metrics have been used to date in upper limb prosthetics research and to guide future research.

## Methods

A scoping review protocol was published on the University of Alberta Education and Research Archive website detailing the methods for this scoping review [13]. The specific aims of this scoping review were: (i) to characterize the visuomotor behaviours of upper limb prosthesis users reported in the literature that have utilized eye tracking technology, (ii) to summarize the eye tracking metrics and variables commonly used to describe behaviours when manipulating a prosthetic hand, and (iii) to identify gaps in the literature and potential areas for future research. Five online databases: Medline, Embase, PsycInfo, ProQuest, and Google Scholar were searched for relevant academic literature published from the dates of their inception until December 1, 2021. The search strategy consisted of terms related to (i) upper limb amputation and prostheses and (ii) assessment of visuomotor behaviour using eye tracking technology. An example of the complete search strategy for Medline is included in the Appendix of the published protocol. Reporting for this scoping review follows the recommendations as outlined by the PRISMA-ScR statement and checklist [14].

Inclusion criteria were peer-reviewed journal articles in which (i) individuals with an upper limb amputation used a prosthesis or individuals with intact arm function used a simulated upper limb prosthesis, (ii) to accomplish an experimental task, (iii) while eye tracking data were collected. Conference papers and dissertations were included, however literature was excluded if the work was preliminary and later published in a peer-reviewed format, or if there were insufficient details to extract the required data. Literature reviews were excluded, as these were found to be summaries of included original papers on other topics, and no review papers were found on this review topic. Studies were excluded if eye tracking was not used as an outcome metric to describe visual behaviour, but rather for control in computer vision. Research on lower limb amputation or prosthesis use, and non-English articles were also excluded.

Title and abstract screening was performed independently by two reviewers (KC and MR). Although literature reviews were excluded from this scoping review, the reference list of two identified review papers were manually searched for relevant literature. This process was to ensure that the original literature was included in the review process. In addition, the following manual searches were conducted in Google Scholar: (i) upper limb prosthesis eye-tracking thesis, (ii) visuomotor control upper limb prosthesis, (iii) cognitive workload artificial limb, (iv) eye tracking artificial limb. All relevant literature was then selected for screening. Two reviewers (KC and MR) completed a full-text review to assess the eligibility of all retained literature. Any conflicts were resolved in consultation with the third reviewer (JH).

Data on the level of amputation, type of prosthetic device, type of eye tracker, primary eye metrics, secondary outcome metrics, experimental task, aims, and key findings were extracted to understand the ways in which eye tracking has been used to evaluate upper limb prosthesis use. Only data pertinent to the research question of this scoping review were reported in the results. Key themes from the literature were identified by grouping together common research goals and experimental methods. Subtopics were subsequently described as related to the overarching theme.

## Results

### Selection of sources of evidence

**Fig. 1:**
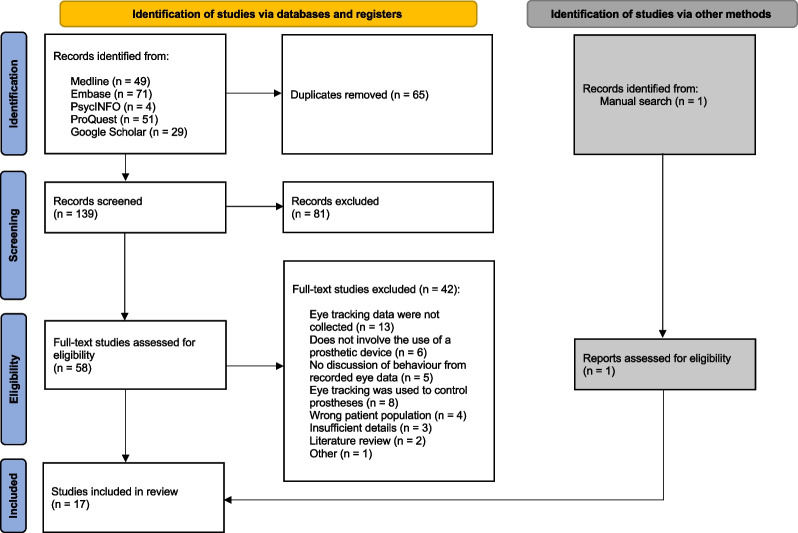
PRISMA flow chart

A database search in Medline, Embase, PsycInfo, ProQuest and Google Scholar produced a total of 204 articles. 65 duplicates were removed, resulting in 139 articles for further screening. After a title and abstract screening, 81 articles were excluded, and a full-text review was conducted on the remaining 58 articles. One article was added manually after reviewing the full text of identified literature. Once the articles were assessed for eligibility, a total of 17 studies were included in the review. The PRISMA diagram (Fig. 1) serves to illustrate this process visually and it includes the details of the reasons for exclusion.

### Characteristics of sources of evidence

Table 1 provides a summary of included study characteristics. All studies quantitatively assessed the behaviour of participants using eye tracking technology. Ten studies consisted of a cross-sectional design [3, 15–23], 3 studies included a repeated-measures study design [21, 24, 25], 2 were crossover studies [26, 27], and 4 were case studies [25, 28–30]. Of the included literature, 1 was a conference paper [23] and 2 were dissertations [16, 20].

**Table 1:** General study characteristics of the included literature

References	Study design	Source of literature	Population	Number of participants
Bouwsema et al. (2012) [17]	Cross-sectional study	Journal article	Upper limb amputation	6
Sobuh et al. (2014) [25]	Repeated-measures study (Experiment 1) Case study (Experiment 2)	Journal article	Intact arm (Experiment 1) Upper limb amputation (Experiment 2)	7 (Experiment 1) 4 (Experiment 2)
Chadwell et al. (2016) [30]	Case study	Journal article	Intact arm Upper limb amputation	1 2
Zhang et al. (2016) [23]	Cross-sectional study	Conference paper	Intact arm	20
Raveh et al. (2017) [27]	Crossover study	Journal article	Intact arm	43
White et al. (2017) [22]	Cross-sectional study	Journal article	Intact arm	20
Chadwell et al. (2018) [18]	Cross-sectional study	Journal article	Intact arm Upper limb amputation	20 20
Parr et al. (2018) [24]	Repeated-measures study	Journal article	Intact arm	21
Raveh et al. (2018) [26]	Crossover study	Journal article	Upper limb amputation	12
Bayani et al. (2019) [15]	Cross-sectional study	Journal article	Intact arm	20
Boser et al. (2019) [16]	Cross-sectional study	Thesis	Upper limb amputation	8
Hebert et al. (2019) [3]	Cross-sectional study	Journal article	Intact arm Upper limb amputation	16 8
Parr et al. (2019) [21]	Cross-sectional study (Experiment 1) Repeated-measures study (Experiment 2)	Journal article	Intact arm	20 (Experiment 1) 24 (Experiment 2)
Zahabi et al. (2019) [29]	Case study	Journal article	Upper limb amputation	1
Kaspersen et al. (2020) [20]	Cross-sectional study	Thesis	Intact arm	6
Chadwell et al. (2021) [19]	Cross-sectional study	Journal article	Upper limb amputation	20
Marasco et al. (2021) [28]	Case study	Journal article	Upper limb amputation	2

### Synthesis of results

**Fig. 2:**
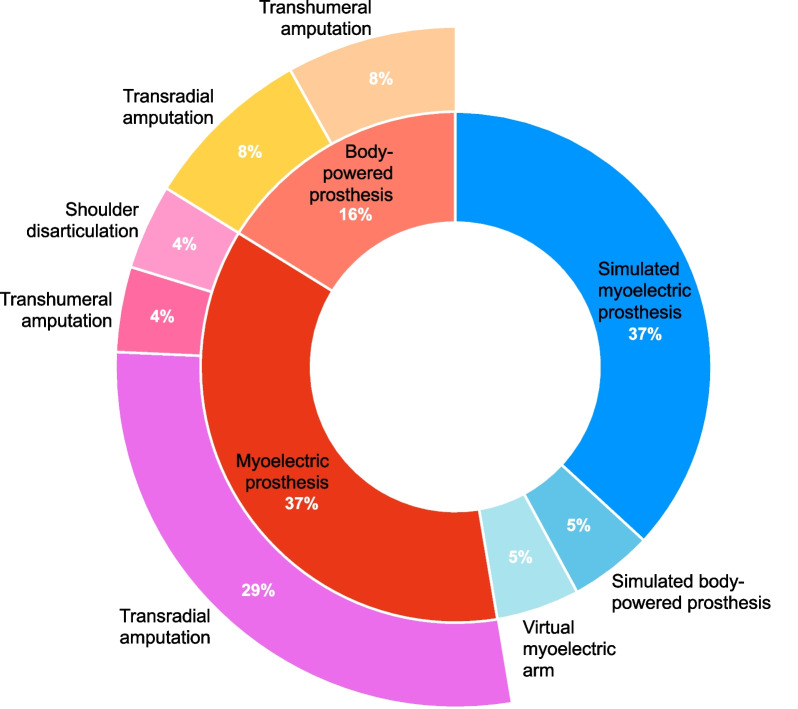
Type of prosthetic device and level of amputation of participants

Participant characteristics were diverse and are summarized in Table 2. A total of 10 studies involved individuals with a limb difference, including 9 studies that tested individuals with transradial amputation [3, 16–19, 25, 26, 29, 30], 3 with transhumeral amputation [3, 16, 28] and one with shoulder disarticulation [28]. Participants with an amputation had a myoelectric prosthesis in 8 studies [3, 17–19, 25, 28–30], while others had a body-powered prosthesis in 2 studies [3, 16]. Nine studies evaluated the visual behaviour of individuals with intact arm function. Of those, 7 studies had individuals perform tasks using a simulated myoelectric prosthesis [21–25, 27, 30]. The simulated device used myoelectric signals to control a terminal device that bypassed the anatomic hand. Alternatively, a simulated body-powered prosthesis was employed in one study [15] and a myoelectrically-controlled virtual reality arm was used in another study [20] with participants who had intact arms. Figure 2 summarizes the type of prosthetic device and level of amputation of participants.

**Table 2:** Participant characteristics of the included literature

References	Sex ratio (M:F)	Age*	Level of amputation	Type of prosthetic device	Number of years using a prosthesis*	Cause of amputation
Bouwsema et al. (2012) [17]	3:3	36 ± 18 (19 to 59)	Transradial	Myoelectric prosthesis	3.8 ± 2.3 (1 to 7) (calculated)	Accident (3), congenital (3), illness (1)
Sobuh et al. (2014) [25]	4:3 (Experiment 1) 3:1 (Experiment 2)	36 ± 10 (26 to 48) (Experiment 1) 49 ± 10 (35 to 56) (Experiment 2)	Transradial	Myoelectric simulator prosthesis (Experiment 1) Myoelectric prosthesis (Experiment 2)	20 ± 13 (2 to 32)	Not reported
Chadwell et al. (2016) [30]	1:0 2:0	21 (44 to 45)	Transradial	Myoelectric simulator prosthesis (1) Myoelectric prosthesis (2)	(1.5 to 35)	Congenital
Zhang et al. (2016) [23]	10:10	23.5 ± 2.36	N/A	Myoelectric simulator prosthesis	N/A	N/A
Raveh et al. (2017) [27]	18:25	26 ± 6.6	N/A	Myoelectric simulator prosthesis	N/A	N/A
White et al. (2017) [22]	10:10	23.5 ± 2.36	N/A	Myoelectric simulator prosthesis	N/A	N/A
Chadwell et al. (2018) [18]	9:11 14:6	43 (23 to 61) 53 (18 to 75)	Transradial	Myoelectric prosthesis	20 (1.5 to 39)	Congenital (11), amputation (9)
Parr et al. (2018) [24]	13:8	25.3 ± 5.05	N/A	Myoelectric simulator prosthesis	N/A	N/A
Raveh et al. (2018) [26]	11:1	65 ± 13^†^	Transradial	Myoelectric prosthesis	15.5 ± 6^†§^	Not reported
Bayani et al. (2019) [15]	10:10	24.7 ± 3.39 (18 to 34)	N/A	Body-powered simulator prosthesis	N/A	N/A
Boser et al. (2019) [16]	7:1	(31 to 64)	Transradial (5) Transhumeral (3)	Body-powered prosthesis (8)	10.6 ± 4.3 (2 to 14) (calculated)	Not reported
Hebert et al. (2019) [3]	8:8 8:0	26 (18 to 43) 45 (30 to 64)	Transradial (5) Transhumeral (3)	Body-powered prosthesis (6), myoelectric prosthesis (1), hybrid hand (1)	11 ± 3.4 (4 to 14) (calculated)	Not reported
Parr et al. (2019) [21]	12:8 (Experiment 1) 12:12 (Experiment 2)	Experiment 1: 25.3 ± 5.05 Experiment 2: 24.4 ± 7.23	N/A	Myoelectric simulator prosthesis	N/A	N/A
Zahabi et al. (2019) [29]	1:0	42	Transradial	Myoelectric prosthesis	2	Accident
Kaspersen et al. (2020) [20]	3:3	26.8 ± 3.1 (23 to 32)	N/A	Myoelectric controlled virtual reality arm	N/A	N/A
Chadwell et al. (2021) [19]	14:6	53 (18 to 75)	Transradial	Myoelectric prosthesis	20 (1.5 to 39)	Congenital (11), amputation (9)
Marasco et al. (2021) [28]	1:1	(38 to 40)	Shoulder disarticulation (1) Transhumeral (1)	Myoelectric prosthesis with touch and kinesthetic feedback tactors	Not reported	Not reported

^*^Values are given as the mean, with or without the standard deviation, in years, with or without the range in parentheses

^†^These data are given as the median with the interquartile range

^§^These data are given in hours per day

Table 3 summarizes the experimental data collection methods. In general, object manipulation tasks were performed with the prosthetic hand while eye tracking data were collected. Tasks involved the Southampton Hand Assessment Procedure (SHAP) [31], as well as tasks modified from the SHAP [21, 24, 25]. Other experimental tasks included a dual task activity [26, 27], a clothespin relocation task [22, 23, 29], a cylinder task [18, 19, 30], a cup transfer task [3, 16, 28], and a pasta box task [3, 16, 28].

**Table 3:** Context and key findings of included studies

Reference	Aims	Type of eye tracker	Eye metrics	Other outcome metrics	Experimental task	Key findings
Bouwsema et al. (2012) [17]	1) To provide a description of prosthetic control and performance 2) To relate clinical outcomes to kinematic measures 3) To identify parameters that characterize the skill level of a prosthesis user	Head-mounted (model RK-826PCI, iScan Online, Inc; Dallas, Texas)	Number of fixations and percent fixation	End point kinematics, joint angles, grasp force control and SHAP^a^	Performed direct and indirect grasping tasks with prosthesis and object manipulation with the non-disabled hand. Objects were solid or compressible. Participants also performed the SHAP^a^	Two types of gaze behaviours were observed: 1) Visual fixations directed towards the object at the start of the trial and maintained; 2) Visual fixations switched repeatedly between the hand and object. Participants who did not use their prosthesis frequently had a higher total number of fixations, lower percent fixation to the object and higher percent fixation to the hand than frequent myoelectric prosthesis users. SHAP^a^ scores did not correlate to measures of gaze behaviour
Sobuh et al. (2014) [25]	1) To characterize the visuomotor behaviours of participants with intact arm function learning to use a prosthesis simulator 2) To compare the visuomotor behaviours of participants with intact arm function and individuals with an upper limb amputation using a prosthesis	Head-mounted (iView X™ HED 2, SenseMotoric Instruments GmbH, Tellow, Germany)	Gaze sequence, percent fixation and number of fixations	Movement time and SHAP^a^	A carton pouring task using the anatomic or the prosthetic hand. The SHAP^a^ was performed as training between testing sessions	When using the prosthesis simulator for carton pouring, gaze was more fixated to the hand in reach and rarely fixated the glass, compared to gaze when using the anatomic hand During manipulation, similar critical areas were focused on regardless of using the anatomic or prosthetic hand. There were significantly greater fixations and a lower SHAP^a^ when using the prosthesis simulator, compared to using the anatomic hand. Training significantly improved SHAP for prosthesis simulator users but had no significant effect on gaze behaviour. Percent fixations, number of fixations during reach, movement times and SHAP scores were similar between subjects with intact arm function and prosthesis users. The number of fixations were higher for prosthesis users during manipulation
Chadwell et al. (2016) [30]	1) To assess factors of the prosthesis control chain, including EMG skill and electrode reliability 2) To evaluate performance, including kinematic and gaze patterns and myoelectric prosthesis usage	Head-mounted (Dikablis Professional, Ergoneers)	Number of fixations, percent fixation and percent of look-ahead fixations	Success of task completion, task duration, aperture onset delay, plateau time during reach to grasp, kinematic variability and symmetry of real-world arm use	Participants began by reaching and grasping for a cylinder rotating it 90°, then placing and releasing it into a tube. If over 80% of trials were successful, the same task was repeated with a smaller diameter cylinder. If less than 80% of trials were successful, the cylinder was placed vertically into a vertical tube	Prosthesis User 1 was able to look ahead to the cylinder and tube, whereas Prosthesis User 2 spent most of the time monitoring the hand and cylinder during reach to grasp. Prosthesis User 1 looked ahead of the hand 76% of the time, while Prosthesis User 2 looked at the hand for over 50% of the time
Zhang et al. (2016) [23]	To compare the cognitive workload of individuals with intact arm function when using a myoelectric prosthesis simulator with direct control or pattern recognition control	Remote (Facelab 5.1, Seeing Machines, Australia)	Percent change in pupil size	Number of clothespins successfully relocated	Clothespin relocation task. Participants moved as many clothespins as possible between the horizontal and vertical bars within a 2-min trial	The pattern recognition group had a greater task performance and lower cognitive load, as shown by a smaller increase in pupil size, than the direct control group. Task performance increased while cognitive workload decreased in later trials
Raveh et al. (2017) [27]	To evaluate the effects of adding vibrotactile feedback on visual attention and performance of individuals with intact arm function using a myoelectric prosthesis simulator in a dual task paradigm	Remote (GP3 Desktop eye tracker, Gazepoint, Canada)	Percent fixation to the screen	Task completion time and percentage of error in secondary task	Dual task involved using the left hand to toggle arrow keys to navigate a virtual car while grasping activities were performed with the prosthesis simulator. Dual tasks were performed with and without vibration	Adding vibrotactile feedback had no effect on visual attention and task performance in a dual task
White et al. (2017) [22]	To compare the usability of direct control and pattern recognition control for individuals with intact arm function using a transradial myoelectric prosthesis simulator	Remote (Facelab 5.1, Seeing Machines, Australia)	Number of pupil size increases per second	Number of clothespins successfully relocated and learning percentage	Clothespin relocation task. Participants moved as many clothespins as possible between the horizontal and vertical bars within a 2-min trial	Participants that used pattern recognition had a lower cognitive workload as shown by fewer increases in pupil size, greater task performance and an improved ability to learn compared to direct control. There was also a trend for task performance to increase across trials for both groups
Chadwell et al. (2018) [18]	1) To report real-world activity of prosthesis users and participants with intact arm function 2) To investigate whether measures of kinematic and gaze behaviour during a goal-directed task correlate to measures of upper limb activity	Head-mounted (Dikablis Professional, Ergoneers)	Percent fixation	Prosthesis wear time, balance of activity between arms, success of task completion, task duration, delay plateau, reach plateau, acceleration temporal variability	Participants began by reaching and grasping for a cylinder rotating it 90°, then placing and releasing it into a tube. If over 80% of trials were successful, the same task was repeated with a smaller diameter cylinder. If less than 80% of trials were successful, the cylinder was placed vertically into a vertical tube	Prosthesis users relied on their anatomical side to perform daily activities whereas participants with intact arm function relied more on both dominant and non-dominant arms. There were no significant correlations between any measures of everyday use and measures of task performance
Parr et al. (2018) [24]	To explore the spatial and temporal disruptions to eye-hand coordination during prosthetic hand use in a fine motor task	Head-mounted (Mobile Eye XG, Applied Science Laboratories, Bedford, MA)	Percent fixation, target locking strategy and gaze shifting	Task completion time	Four coins on a board were sequentially picked up from right to left and placed in a jar located in the centre of the board. The task was first performed with the anatomic hand then the prosthetic hand	When using the prosthetic hand, significantly greater visual attention was directed towards the hand and coin and less visual attention to other target areas, when compared to the anatomic hand. In all phases, more time was spent fixating the hand than the target when using the prosthetic hand. There was a significant delay for the eyes to disengage from the current target and shift to the next movement
Raveh et al. (2018) [26]	To evaluate the effects of adding vibrotactile feedback to myoelectric prostheses on visual attention and performance in a dual task paradigm	Remote (GP3 Desktop eye tracker, Gazepoint, Canada)	Number of fixations to the hand and percent fixation to the screen	Task completion time and percentage of error in secondary task	Dual task involved using the left hand to toggle arrow keys to navigate a virtual car while grasping activities were performed with the prosthesis. Dual tasks were performed with and without vibration	Adding vibrotactile feedback reduced task performance time in a dual task activity performed by myoelectric prosthesis users. There was no effect of adding vibrotactile feedback on gaze behaviour
Bayani et al. (2019) [15]	To identify gaze strategies that develop implicitly during matched and mismatched limb training during action observation	Head-mounted (Pupil Labs, binocular, Berlin, Germany)	Percent fixation	Movement time, number of errors, type of errors, peak height, peak velocity, peak lateral trunk movement, variability in lateral trunk movement and smoothness	The task involved reaching and grasping for a disc and transporting it over a barrier to be placed in an open slot. Participants watched an instruction video performed either by an actor with a body-powered prosthesis (matched) or with the anatomic limb (mismatched). After watching the video, participants performed the task with the prosthetic hand	In the mismatched group, gaze fixations were directed towards the start and endpoints of the action, whereas for the matched group, gaze was focussed on the path of the prosthesis and the shoulders. With matched action observation, the allocation of gaze shifted from the start and end locations towards monitoring the trajectory of the prosthesis across trials. There was a progressive improvement in motor control in the matched group
Boser et al. (2019) [16]	To characterize the visuomotor behaviour of transradial and transhumeral body-powered prosthesis users	Head-mounted (Dikablis Professional, Ergoneers)	Percent fixation, target locking strategy, number of fixations and eye latencies	Task completion time, hand distance travelled, hand trajectory variability, number of movement units, peak hand velocity, percent to peak hand velocity, percent to peak hand deceleration, percent to peak grip aperture	A cup transfer task involved moving two cups sequentially across a partition to two target locations then returning the cups to their starting locations. A pasta task involved moving a pasta box from a starting location on the side of the body to a centre shelf, then around a barrier to a second higher shelf, then to its starting location	Transradial body-powered prosthesis users had longer task completion times, increased fixations to their prosthetic hand during reach and transport phases, and movements that were not as smooth compared to individuals with intact arm function. Look-ahead fixations to the drop-off target were within a normative range. Transhumeral body-powered prosthesis users had similar movements as transradial body-powered prosthesis users, however increased fixations to the hand in transport prevented the ability to look ahead to the drop-off target. In the cup transfer task, there were longer fixations to the terminal device during reach than the pasta task
Hebert et al. (2019) [3]	To determine whether different tasks performed by prosthesis users would result in different visuomotor behaviours	Head-mounted (Dikablis Professional, Ergoneers)	Percent fixation and eye latencies	Movement time and upper body range of motion	A cup transfer task involved moving two cups sequentially across a partition to two target locations then returning the cups to their starting locations. A pasta task involved moving a pasta box from a starting location on the side of the body to a centre shelf, then around a barrier to a second higher shelf, then to its starting location	The cup transfer task required more visual attention to the hand than the pasta task during reach and transport phases, likely due to the risk of spilling the contents of the cup. In both tasks, users had prolonged eye latencies and less fixation on the current target compared to a normative group
Parr et al. (2019) [21]	1) To explore the spatial and temporal disruptions to eye-hand coordination during prosthetic hand use in a fine motor task 2) To explore the efficacy of a novel gaze training intervention on prosthetic hand skill learning and retention compared to movement training	Head-mounted (Mobile Eye XG, Applied Science Laboratories, Bedford, MA)	Target locking strategy and gaze shifting	Task completion time, number of errors, alpha power and high alpha connectivity	Experiment 1: picking up a jar filled with water over a barrier to a location on the other side of the board. The task was performed with the anatomic hand first followed by the prosthetic hand Experiment 2: Four coins on a board were sequentially picked up from right to left and placed in a jar located in the centre of the board. A tea making task involved placing a mug on a place mat, adding and then stirring contents with a spoon	With the prosthetic hand, participants focused significantly more on the hand and had a time delay to disengage visual attention in all phases of the jar task. There was also a global decrease in alpha power, indicating increased cortical activation and mental effort. Gaze training increased fixations to the target and speed of gaze shifts, reduced performance time, and improved neural efficiency compared to movement training. These improvements were transferred to a more complex tea-making task. Target locking strategy and faster gaze shifting were significant predictors of T7-Fz connectivity (indicates less conscious control) at retention and delayed retention with gaze training
Zahabi et al. (2019) [29]	To assess the validity of using a cognitive model to assess the mental workload of upper limb prosthesis use under direct control and pattern recognition control	Remote (Facelab 5.1, Seeing Machines, Australia)	Pupil size	Task completion time and number of clothespins successfully relocated	A single subject performed the clothespin relocation task with two different control modes on two separate days. A cognitive performance model was then constructed to compare the demands of using different control modes	Significantly more clothespins were moved with pattern recognition compared to direct control. Using pattern recognition also resulted in a smaller pupil size, indicating lower cognitive load. The cognitive model indicated that there were fewer cognitive and motor operators with pattern recognition than direct control and no difference in the number of perceptual operators. The model underestimated task completion times
Kaspersen et al. (2020) [20]	To evaluate the feasibility of using an eye tracker to quantify the sense of agency towards a virtual limb controlled using myoelectric pattern recognition	Head-mounted (Tobii Pro Glasses 2, Tobii AB, Stockholm, Sweden)	Duration of fixations and number of fixations	Reaction time	Four onscreen virtual reality arms were controlled using myoelectric control. Different levels of noise were introduced to randomly reclassify movements to 3 of the 4 virtual arms, making them less controllable. Random arms would flash red at two random time points during each trial and participants were instructed to press a key when they detected a red flash	Two types of gaze behaviours were observed. Participants either fixated on the centre of the screen and used peripheral vision to detect red flashes, or they moved around to fixate on each quadrant. Significantly more time was spent fixating on the most controllable virtual arm, however noise level did not affect the time taken to react to a red flash. Results suggest that visual attention is directed to the virtual arm that provides the best experience of agency to the participant
Chadwell et al. (2021) [19]	To establish the relative impact of control factors (signal acquisition, signal generation and device response) on user functionality (task performance, kinematics and gaze behaviour) and everyday prosthesis usage	Head-mounted (Dikablis Professional, Ergoneers)	Percent fixation and number of fixations	EMG signal, reaction time, number of undesired activations, electromechanical delay, number of successes, task completion time, reach aperture plateau, movement variability, prosthesis wear time and balance of activity between arms	Participants began by reaching and grasping for a cylinder rotating it 90°, then placing and releasing it into a tube. If over 80% of trials were successful, the same task was repeated with a smaller diameter cylinder. If less than 80% of trials were successful, the cylinder was placed vertically into a vertical tube	A higher number of unwanted EMG activations was significantly correlated to lower success rate, longer task duration, higher temporal kinematic variability, increased fixations to the hand, decreased fixations to grasp critical areas during reach to grasp and increased gaze switches. Longer electromechanical delay was significantly correlated to shorter task duration, shorter length of aperture plateau, decreased fixations to the hand during transport, increased fixations to location critical areas during transport, fewer gaze switches and longer prosthesis wear time
Marasco et al. (2021) [28]	To quantify the performance of individuals who received targeted sensory and motor reinnnervation using metrics such as visual attention, cognitive demand, fine motor dexterity and ownership	Head-mounted (Pupil Labs, binocular, Berlin, Germany)	Percent fixation	Prosthesis Efficiency and Profitability, Dynamic Prosthesis Incorporation, Grasping Relative Index of Performance, Adaptation rate	A cup transfer task involved moving two cups sequentially across a partition to two target locations then returning the cups to their starting locations. A pasta task involved moving a pasta box from a starting location on the side of the body to a centre shelf, then around a barrier to a second higher shelf, then to its starting location	Providing touch and kinesthetic feedback to prosthesis users with targeted sensory and motor reinnervation reduced fixations to the hand in reach and transport phases, and increased fixations to the next target location. Overall, the integration of bidirectional control allowed users to adopt more natural behaviours

^a^Southampton Hand Assessment Procedure

**Fig. 3:**
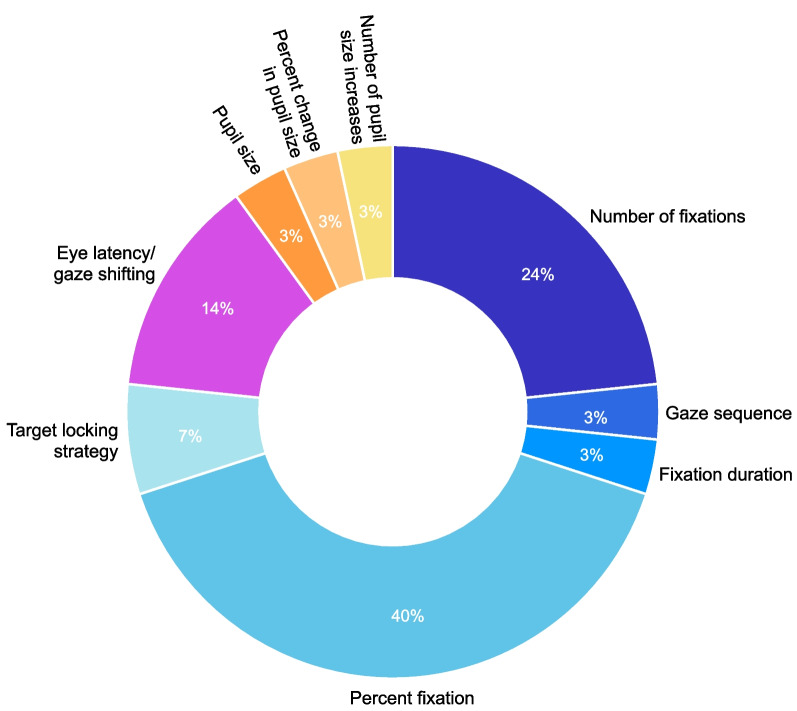
Eye metrics reported in the literature to evaluate prosthetic visuomotor behaviour

To characterize the visual behaviours of participants, several eye metrics were recorded and are summarized in Table 3. The direction of gaze was recorded to determine the location of overt visual attention [4]. Key areas of interest (AOI) were defined in each study that were relevant to the specific task demands. Since areas unrelated to the goal of the task are rarely fixated [8–10], areas such as the hand, start location, end location and objects being manipulated were usually defined as AOIs. Eye metrics used to describe prosthetic visuomotor behaviour included both spatial and temporal information, such as when and where someone was looking. In the spatial domain, these metrics included the number of fixations, gaze sequence, duration of fixation, percent fixation and target locking strategy (TLS), and in the temporal domain, these metrics included eye latency measures. Figure 3 presents the distribution of eye metrics that are reported in the literature.

To describe the spatial allocation of gaze, the number of fixations referred to the frequency of visual fixations to defined AOIs and was used to indicate how often the gaze switched between the hand and the target to monitor the trajectory of the prosthetic hand [16, 17, 19, 20, 25, 26, 30]. While the number of fixations only reported the frequency of fixations, gaze sequence provided additional information about the order of visual fixations to AOIs in addition to the location, and demonstrated the pattern in which visual fixations occurred throughout each trial [25]. Fixation duration referred to the absolute time in seconds that the gaze fixated onto an AOI [20]. This metric is useful in comparing the absolute amount of attention towards an AOI during fixed time trials. However, for trials that are variable in length, percent fixation was generally used as a preferred metric to compare the relative allocation of visual attention to the hand and target areas. Percent fixation was defined as the amount of each phase that was spent fixating a given AOI and was represented as a percentage of each phase [3, 15–19, 24–28, 30]. Additionally, TLS was the difference between the percentage of time spent fixating the target and the hand [21, 24]. Using this metric, a more positive value reflected more time spent fixating the target, whereas a more negative value indicated more time spent fixating the hand [21, 24]. A score close to zero represented a gaze switching strategy, in which equal amounts of time were spent fixating the target and the hand [21, 24]. Together, these metrics provide a detailed description of the efficiency of gaze control and where upper limb prosthesis users predominantly focus their attention.

To describe the temporal relationship between eye and hand movements, eye latency measures were defined as the time in seconds that the eyes precede or follow the movement of the hand [3, 16, 21, 24]. Eye arrival latency described the timing in which the eyes fixated the target location before the arrival of the hand [2]. Eye leaving latency referred to the timing to disengage gaze from the target of interest [2]. Note that Parr et al. [21, 24] described the same metric which they referred to as gaze shifting. A negative time indicated that the eyes were ahead of the hand, while a positive time reflected the time in which the eyes lagged behind the hand. This metric is useful in understanding human–machine interactions, as it uncovers the temporal dynamics between the location of visual attention and the location of the prosthetic hand and objects.

Additional eye metrics described in the literature measured pupil dilation to describe the cognitive workload associated with prosthetic use. These metrics included pupil size, percent change in pupil size, and number of pupil size increases. Pupil size was measured as the average diameter of the pupil in millimeters during a trial [29]. Percent change in pupil size was measured as the difference between the maximum pupil diameter and minimum pupil diameter at baseline, relative to the baseline pupil diameter [23]. This metric did not account for differences in individual pupil sizes, which may not be appropriate for a between-subject design [23]. Number of pupil size increases, therefore, measured the number of times that the pupil diameter increased per second [22], as an index of cognitive activity [32] and was less susceptible to environmental factors [22].

### Thematic synthesis

Three main themes were identified in the reviewed literature: (i) general visuomotor behaviours of prosthesis users, (ii) different experimental conditions across research groups, and (iii) changes in gaze behaviour in response to various factors. In addition, other exploratory areas of eye tracking in prosthesis research were uncovered in the literature that do not coincide with the aforementioned themes. Early studies aimed to characterize the visuomotor behaviours of upper limb prosthesis users. Across research studies, differences in experimental protocols revealed significant differences in these behaviours. Later work began to use eye tracking as an outcome measure to assess the effectiveness of novel prostheses interventions. Lastly, two additional areas of prosthetic behaviour, cognitive workload and sense of agency, used eye metrics for evaluation.

## Discussion

### Characteristics of visuomotor behaviours of upper limb prosthesis users

#### Eye-hand coordination of prosthesis users

When reaching and grasping with the anatomic limb, the eyes tend to fixate on a target ahead of the hand movement and maintain fixation on that target while executing the task. Thus the eyes rarely fixate on the hand [8]. Prosthesis users, however, have been shown to exhibit different patterns of visuomotor behaviour. The literature consistently demonstrated that when reaching and grasping for an object with the prosthetic hand, the eyes fixated more towards the hand and less towards the target [3, 16, 17, 21, 24, 25]. Other studies further revealed increased fixations to the prosthetic hand when transporting an object [3, 16, 21, 24]. The reliance on vision to monitor the hand was described to be due to grip insecurity and deficits in sensory feedback [3, 16, 21, 24, 30]. In addition, a gaze switching strategy was identified, in which prosthesis users continuously switched between visually monitoring the hand and the target, as indicated by an increased number of fixations [17, 25] and a low TLS score [21, 24]. Some studies further showed that there was a significant delay for the eyes to disengage from the current target and shift to the next target when manipulating objects with a prosthesis [3, 16, 21, 24].

Disruptions to normative eye-hand coordination that have been highlighted in the literature are reflective of an increased visual dependency to compensate for difficulties associated with prosthetic use. The increased attentional demand that is needed to visually monitor the activity of the prosthesis can be one factor that is cognitively demanding and is often reported to be the primary reason for device dissatisfaction and rejection [33, 34]. Since haptic and proprioceptive feedback are lost with amputation, it is likely that vision is used as a feedback mechanism to ensure a stable grip [35]. This reliance on vision prevents the eyes from looking ahead towards target objects to plan for upcoming actions [21, 24]. Therefore, adjustments to the hand need to be actively controlled and can place a high cognitive demand on the user [21].

#### Gaze behaviour as an indicator of skill level

The development of eye-hand coordination during human development phases is well-defined. In the initial stages of motor learning, vision is used as a feedback mechanism to monitor the actions of the hands [36]. As skill develops, vision shifts to be predictive of hand movements and the eyes are able to look ahead towards the target [36]. Presumably then, the reliance on vision to monitor the prosthesis would decrease with experience and more highly skilled prosthesis users would demonstrate eye-hand coordination typically observed in individuals with intact arm function. Some studies [17, 24, 25] have suggested that more experienced prosthesis users behave similarly to individuals with intact limbs, however findings have been inconsistent.

Parr et al. [24] suggested that prosthetic eye and hand movements remain temporally coupled. Results of their study revealed that the timing of gaze shifts was a significant predictor of task performance time, therefore the ability to shift vision away from the hand towards the target resulted in faster movements [24]. Another study however, showed that the gaze strategies of experienced prosthesis users are highly variable [17]. While some experienced users fixated predominantly towards the target, others switched between monitoring the hand and the target. Measures used to describe the spatial allocation of visual attention, including percent fixation and number of fixations, thus were not related to skill level [17] or everyday usage [18]. Despite clear differences in the visuomotor behaviours of individuals with intact arm function and prosthesis users, it is unclear from the findings to date, whether behaviours that more closely resemble normative eye-hand coordination are indicative of a higher prosthesis skill level.

Disruptions to the development of typical patterns of eye-hand coordination reported in upper limb prosthesis users can also be attributed to the unreliability of prosthetic devices. A multitude of factors, such as electrode shift, electrode impendence, and fatigue affect the reliability of myoelectric control [37], preventing typical sensorimotor mapping rules from developing [38]. As a result, it is likely that vision maintains fixated on the prosthetic hand, even as skill progresses to compensate for the unpredictable control. Future work should investigate the relationship between gaze behaviour and skill level, and whether addressing the unpredictability of myoelectric control can alleviate demand on the visual system.

### Experimental conditions can influence visuomotor behaviours

The visuomotor behaviours of prosthesis users has been characterized across multiple research groups that have employed different experimental conditions. The general findings were largely in concordance with one another, apart from a few studies that indicated that the type of prosthetic device and experimental task produced notable differences in prosthetic behaviour.

#### Type of prosthetic device

Participants with intact arm function using a simulated prosthesis and individuals with amputation using a prosthesis have demonstrated similar eye and hand movement patterns when using a prosthetic device. Sobuh et al. [25] demonstrated that participants using a simulated myoelectric prosthesis and experienced myoelectric prosthesis users had similar gaze fixations, movement times, and SHAP scores. In addition to similarities in visuomotor behaviours between the two groups, the compensatory movement patterns of individuals using a simulated device have also been shown to be similar to individuals with an upper limb amputation when using a myoelectric prosthetic device [39]. Although this evidence suggests that the use of a simulator prosthesis is an acceptable proxy for studying upper limb prosthesis use, considerations should be made concerning the translatability of results to prosthesis users. The attachment of a terminal device to a simulated prosthesis presents several challenges. The position of the terminal device can affect the centre of mass or obscure the view of the prosthesis [40], which may affect visuomotor behaviour. In addition, the long-term use of a simulated prosthetic device has yet to be explored, therefore it is unknown whether visuomotor behaviours observed during initial testing sessions are representative of long-term device use for prosthesis users. However, recruiting participants with intact arm function as an alternative to upper limb prosthesis users allows a larger sample size to increase the statistical power of the results. Novice users are also assumed to have no experience with operating a prosthetic device, whereas prosthesis users typically have varied experience levels [17]. Using naïve participants allows researchers to control for level of experience when evaluating novel research interventions.

In general, myoelectric and body-powered prosthesis users demonstrated similar visuomotor behaviours. Both myoelectric and body-powered prosthesis users took longer to complete tasks with the prosthetic hand compared to the anatomical hand and a disproportionate amount of time was spent fixating the prosthesis when reaching and transporting objects [3, 16, 25]. However, some notable differences in gaze behaviour were observed that were unique to body-powered prosthetic use. A gaze switching strategy was not evident in transradial body-powered prosthesis users likely due to the mechanics of these types of devices [16]. Unlike the unreliable nature of myoelectric control that may cause the prosthetic hand to unexpectedly open, a voluntary open hook was used, which remained closed on objects once grasped. Since a relatively stable grasp can be achieved with this type of terminal device, vision was not required to monitor the prosthetic hand. This device, along with an intact elbow providing some proprioceptive feedback, enabled users to look ahead towards the drop-off target within a normative range of behaviour [16]. However, transhumeral body-powered prosthesis users faced an additional visual demand with increased fixations to the terminal device in transport that prevented the ability to look ahead to the drop-off target [16]. Researchers should therefore consider how the type of prosthetic device as well as the level of amputation might affect overall research outcomes.

#### Experimental task conditions

Across research groups, different experimental tasks were employed to study the visuomotor behaviours of prosthesis users. Interestingly, despite these differences, the observed gaze behaviours were consistent across studies [16, 17, 21, 24, 25]. This finding is in accordance with non-disabled eye-hand coordination studies that have also shown remarkable agreement across various functional tasks, which has led to important generalizations about human behaviour [8–11]. The majority of studies included in this review paper involved relatively simple tasks that were performed in a seated position and largely limited to the task space directly in front of the participant [17–19, 24, 25, 30]. Individuals with upper limb amputation are known to use their prosthesis for a broad range of activities of daily living (ADL). The prosthesis is most frequently used in bimanual tasks to assist the intact limb [41]. In addition to desk procedures, similar to the experimental tasks of the included studies, common ADLs involving prosthetic use include housework, shopping, eating and cooking [42]. Therefore, the limited movements of these experimental tasks may, not be generalizable to prosthesis user functionality in everyday tasks [18].

Only one study specifically addressed the different task demands of two goal-oriented object manipulation tasks on the visuomotor system [3]. A pasta box task and a cup transfer task were developed to represent daily activities [43] and validated to quantify gaze behaviour [2]. The pasta box task required gross movements to transport a pasta box from various shelf heights, whereas the cup transfer task involved transporting compliant cups filled with beads [43]. The different task demands revealed differences in visuomotor compensatory strategies, in which the cup transfer task required more visual attention to the hand than the pasta box task during reach and transport phases. Since the cups are deformable, the authors explained that visual attention towards the hand was likely a cautious strategy to ensure contents of the cup were not spilled [3]. Therefore, the cup transfer task was applicable in evaluating gaze strategy in relation to grasping skills (including maintaining grasp during transport), whereas, the pasta box task challenged users in various planes of movement that required users to adapt their visual behaviours [3]. These findings highlight the importance of considering the task being used when measuring visual behaviour. Experimental conditions should include scenarios that represent activities of daily living, as lab-based tasks may not be representative of eye movement behaviours in the real world [44].

In addition to experimental task selection, the type of eye tracker should also be considered in the experimental design. Remote eye trackers restrict head and body movements to the area directly in front of the eye tracker for reliable gaze recording. Such postural constraints have been shown to affect gaze behaviour, specifically, the velocity of saccades was shown to increase when the head was restricted [45]. When the head was unrestrained and could move freely in the recording area of the remote eye tracker, data loss and spatial accuracy errors were apparent [46]. Head-mounted eye trackers, on the other hand, maintain ecological validity and allow for natural eye movements. In accompanying more functional tasks of daily living, researchers should consider preferentially using a head-mounted eye tracker to allow for natural eye, head, and hand movements that are representative of real-world scenarios.

### Responsiveness of eye metrics to various factors

To date, the use of eye tracking technology has enabled researchers to establish a characteristic visuomotor behaviour of upper limb prosthesis users that differs from the behaviours of individuals with intact arm function. However, the question remains whether eye tracking metrics are sensitive enough to effectively assess functional improvements, such as to reduce the visual demand associated with prosthetic use. The following section will explore the effects of control systems, sensory feedback, and training on gaze behaviour and provide evidence that eye metrics are sensitive to various factors.

#### Prosthetic control chain

Myoelectric prostheses are challenging to control as there are many factors involved in controlling a prosthesis, which can explain why vision is drawn towards the prosthetic hand. Minimizing uncertainty in the controller can improve grasp performance [35] and also has the potential to reduce visual attention towards the hand. Research on the prosthetic control chain has investigated factors such as signal generation, signal acquisition, and device response [18, 19, 30].

To understand which control factors may contribute to improving user functionality, Chadwell et al. [18, 19, 30] investigated the relationships between each of these control factors and measures of functionality and everyday prosthesis usage. Their results showed that gaze behaviour was significantly disrupted by mechanical issues, such as unpredictability and electromechanical delay, and was not related to skill in controlling the electromyography (EMG) signal [19]. Unpredictability, as defined by a higher number of unwanted EMG activations, was significantly correlated to lower success rate, longer task duration, higher temporal kinematic variability, increased fixations to the hand, decreased fixations to target areas during reach to grasp, and increased gaze switches [19]. Longer electromechanical delay was also related to improved performance, such as shorter task duration, shorter length of aperture plateau, decreased fixations to the hand during transport, increased fixations to target areas during transport, fewer gaze switches, and longer prosthesis wear time [19]. This finding was in contrast to their hypotheses and is counterintuitive. The effect of device delay on user performance is not well understood, however the authors speculate that increased mechanical delays may actually reduce undesired activations of the prosthesis. Additional research is needed to investigate the interactions between mechanical issues and their influence on visuomotor behaviours.

The addition of a prosthesis introduces unpredictable control, which appears to drive the dependency on vision to monitor the hand. Gregori et al. [47] revealed that when individuals with a transradial amputation were asked to grasp and manipulate objects with their missing limb rather than their prosthesis, they demonstrated similar visuomotor behaviours as individuals with intact arm function. Therefore, it is likely that the complexities of translating muscle signals into actions of the prosthesis is what introduces the disruptions to gaze behaviours. Together, these findings point towards a need to address factors affecting control reliability and future work should consider including eye tracking as an outcome measure to assess the usability of novel control systems.

#### Sensory feedback systems

The integration of sensory feedback systems in myoelectric prostheses have shown promise in improving performance [48], and some researchers have investigated whether adding supplementary feedback can reduce the visual attention on the hand. One research group studied the potential of adding supplementary sensory feedback to normalize gaze behaviour [26, 27]. In their testing paradigm, vibrotactile feedback had no effect on gaze behaviour in participants using a simulated myoelectric prosthesis [27] or myoelectric prosthesis users [26]. A dual-task was used to test differences in gaze behaviour, whereby performance on a primary task assessed the amount of cognitive effort exerted for that task, while performance on a secondary task assessed the remaining cognitive capacity [49]. This testing paradigm may not have been appropriate for discriminating differences between conditions with and without feedback, as the authors suggested that the secondary task was too simple and may not have adequately challenged the user [26, 27]. In addition, the location of gaze may not discern attentional demand in a dual task, as the locus of attention can differ from the location of gaze, particularly when information is processed through the peripheral vision [4]. In the included studies, the effectiveness of sensory feedback was not tested over multiple training sessions. Markovic et al [50] demonstrated that the relevance of feedback is related to the prosthesis user’s experience level. Feedback was only beneficial in reducing task completion time after subjects trained over multiple sessions to learn to control a prosthesis [50]. Therefore, the integration of supplemental feedback into the motor control loop may require time before feedback becomes useful in reducing the reliance on visual feedback.

Although prosthesis users lack touch and proprioceptive feedback, incidental feedback is relayed to the user through visual and auditory cues. Many studies have compared supplementary feedback to baseline conditions where vision is occluded. This method provides a means of isolating the effects of supplementary sensory feedback and has been shown to be useful in controlling grip aperture, grasping force, joint position, and object size and stiffness discrimination [51]. However, few of these studies have considered if supplementary feedback provides additional benefits in the presence of visual feedback. Sensinger and Dosen [51] recommended that the modality of feedback should be purposeful in relaying variables to the user that are not redundant with the information that is already provided through visual feedback. There is currently an absence of evidence to determine whether supplementary feedback is beneficial in reducing the reliance of vision to monitor the prosthesis.

However, one study [28] has demonstrated that restoring sensory feedback through natural channels can restore typical patterns of visuomotor behaviour. Targeted reinnervation is a surgical technique that provides intuitive bidirectional control by rewiring nerves from the amputated limb to new target sites in the muscles and skin [52]. Tactors were integrated into the prosthesis to provide physiologically matched touch and kinesthetic feedback to the reinnervated skin and muscle sites [28]. Compared to no feedback, providing kinesthetic and tactile feedback reduced fixations to the prosthetic hand when reaching and transporting objects, and increased visual fixations to the next target location [28]. In this study, eye tracking was shown to objectively assess visuomotor behaviours of prosthesis users during a goal-directed task and was sensitive to detect functional changes in response to a novel sensory feedback intervention. Given these findings, future work should incorporate the use of eye tracking to ascertain the ability for sensory feedback systems to reduce the burden on the visuomotor system.

#### Training interventions

The goal of training is to improve functional outcomes of prosthesis users. Although training can improve speed [53] and performance [25], less is known about the effects of prosthetic training on gaze behaviour. The reviewed literature reveals that there are notable differences in the way in which persons learning to use an upper limb prosthesis are trained, which can affect functional outcomes. For example, different implicit gaze strategies were developed when observing an instructor with amputation demonstrate a task using a body-powered prosthesis, as opposed to an instructor with intact arm function who demonstrated the same task using their anatomic limb [15]. Those who were trained with observing the body-powered prosthesis user focussed primarily on the path of the prosthesis and the shoulders, which may have facilitated kinematic improvements when executing the task [15]. Therefore, guiding users to adopt gaze fixation patterns that are task specific may be beneficial in promoting more efficient motor learning during prosthesis use.

In fact, one study [21] explored the use of gaze training to teach novice prosthesis users. Gaze training is an implicit learning strategy that teaches users to adopt eye movement behaviours that are similar to expert users by encouraging users to look ahead towards the target instead of monitoring the hand. In contrast, traditional movement training instructs users on how to move their limbs, which can place a high attentional demand on using the prosthetic device. Gaze training resulted in greater fixations towards the target, shorter latencies for the eyes to shift to the next target and shorter performance times than movement training [21]. Not only did gaze training reduce the attentional demand that is associated with prosthetic hand use, but also the cognitive demand, as measured by EEG connectivity between T7 and Fz regions. The interaction between motor planning (Fz) and verbal-analytical (T7) regions of the brain were reduced with training, which reflected a reduction in conscious movement control [21]. Therefore, encouraging users to fixate the target improved neural efficiency and the usability of the prosthetic device. Importantly, those who received traditional movement training demonstrated no improvement in gaze behaviour, despite significant improvements in performance time [21]. The authors indicated that prosthesis users appear to maintain an overreliance on vision to compensate for prosthesis unpredictability and may not be capable of achieving feedforward gaze control through repeated practice. These results suggest that specific focus should be placed on teaching cognitive strategies during training that are aimed at reducing visual attention to improve functional outcomes.

### Other uses of eye tracking to evaluate prosthetic behaviour

#### Pupil dilation used to measure cognitive load

Eye tracking has also been applied to measure changes in pupil size during prosthesis use to assess cognitive workload. Previous studies have attributed visual attention towards the hand as a proxy for cognitive demand but did not provide a direct measure of this experience [24, 25]. Pupil dilation could provide a direct, yet relatively unobtrusive method of measuring the cognitive workload associated with controlling a prosthesis. The pupils have been shown to dilate during mentally demanding activities, such as thinking and memory recall, and return to baseline following the mental task [54]. The benefit of measuring pupil dilation is that it is an objective and unbiased measure. Changes in pupil size are not voluntarily controlled by the user [55] and an eye tracker allows for natural movements. The drawback however is that pupillary size can respond to changes in light and can be confounded by other physiological factors such as anxiety and stress [56]. Other physiological measures (e.g. electroencephalography) require laborious experimental setup that can be obtrusive to the individual, can hinder their functional abilities, and may be susceptible to movement artefacts [57].

Measures of pupil diameter have been commonly used to measure cognitive load in the general population [56]. Recently, these metrics have also been applied to quantify cognitive load in the context of prosthesis use. Cognitive load was evaluated to compare the usability of two different control schemes: direct control and pattern recognition. Pattern recognition was determined to be less cognitively demanding than direct control, as indicated by a smaller change in pupil size [23] and fewer pupil size increases [22]. Since direct control requires additional mental steps to switch between control modes, the authors concluded that pattern recognition was more intuitive to use [22, 23]. In addition, task performance increased across trials for both control modes [22, 23], however pattern recognition was easier to learn and led to superior performance compared to direct control [22]. Zahabi et al. [29] further performed a cognitive modelling study using the average pupil size to predict the cognitive load of the two different control modes. Their predictions corroborated with the findings of previous studies [22, 23] and indicated that fewer cognitive processes and motor commands were required for the pattern recognition control, making it less cognitively demanding than direct control. Evidently, pupil dilation shows promise as a means to non-invasively measure cognitive workload. Future work should address the reliability and validity of pupil dilations in quantifying cognitive workload of prosthesis users.

#### Fixation duration used to measure sense of agency

The sense of agency towards a prosthetic limb can be described as the experience of voluntary control over a prosthetic limb to reliability perform movements as intended by the user [58]. This experience of agency is essential for the prosthesis to be embodied as part of one’s own body [58]. Typically, to assess agency, explicit and implicit measures have been defined [59]. For example, questionnaires are used to explicitly report the experience of an experiment, but self-report relies on users to retrospectively recall the experiment and phrasing of the questions can influence outcome measures [59]. An example of an implicit measure is the intentional binding effect, in which the perceived time interval between a voluntary action and a resulting cue appear shorter than when the action is involuntary [59].

One preliminary feasibility study investigated the use of eye tracking to measure the sense of agency towards a prosthetic limb. Using gaze behaviour and reaction time in a simple detection task was shown to be feasible in assessing the perceived sense of agency [20]. Participants in this study simultaneously controlled four virtual onscreen arms that portrayed active grasp using EMG signals. Different noise levels were introduced to these virtual arms, to randomly reclassify the intended movements. Findings demonstrated that participants spent more time fixating on myoelectric-controlled virtual arms that were most controllable and corresponded to the actual movement intent recorded by EMG signals (i.e. no random noise) [20]. The authors suggested that visual attention is directed towards the virtual arm that provides the best sense of agency [20]. Although there was a significant difference in the allocation of visual attention to different virtual arms, the translatability of such evidence should be considered during functional tasks where visual monitoring of the prosthetic hand is undesirable [3, 16, 17, 21, 24, 25]. Visual and proprioceptive cues about our bodily movements are needed to perceive control over one’s voluntary actions [60]. In this experimental design, where a virtual arm was controlled, vision was the only mode of feedback, as participants did not receive proprioception from a physical arm to perform a functional task. It is therefore reasonable that participants fixated the most controllable virtual arms, however, the sense of agency cannot be confirmed with vision alone. To the best of our knowledge, eye tracking has not been otherwise implemented in prostheses research to measure the experience of agency. As this study is very preliminary, future work is needed to understand the role of vision towards the sense of agency and to test the validity of fixation duration as a metric to evaluate prosthetic agency.

### Limitations and future work

Although this scoping review has compiled a collection of studies that have used eye tracking to assess the visuomotor behaviours of upper limb prosthesis users, we have not provided the reader with a critical discussion around the eye tracking technology itself. Many of the included studies provided limited details on their eye tracking setup. As such, future work should consider providing additional details on eye data collection, processing, and analysis methods for a more comprehensive insight into the technology. A review of the eye tracking technology applied to a wider population would serve useful in highlighting the limitations of eye trackers and the implications in understanding visuomotor behaviours.

To describe the visuomotor behaviours of prosthesis users, researchers have utilized visual fixations to infer cognitive effort. However, this metric only captures overt visual attention and does not encapsulate all of the cognitive, physical and emotional workload characteristics experienced by prosthesis users. Presently, only pupil dilations have been explored as an eye metric to directly quantify the cognitive workload of prosthesis users, although additional work is needed to verify the validity of this metric. Recent work has shown promise in developing a valid measure of cognitive workload and using eye tracking to correlate workload with visual attention [61]. Fixations towards the hand were related to a multitude of factors that represent mental workload, such as mental demands, physical demands, visual demands, conscious processing, frustration, etc. [61] This prosthesis user-specific workload measure may serve useful in future research to better understand the multifaceted challenges of prosthetic use.

An additional eye metric that has not yet been explored in prosthetics research is blink rate. Eye blink metrics have revealed cognitive processes in non-disabled populations, as these are known to be dependent on levels of mental activity [62]. In healthy humans, blinks occur around 15–20 times per minute [63] and have been shown to be reduced in mentally demanding tasks or when engagement levels were high [64]. Therefore, eye blink metrics may potentially provide researchers with another marker of cognitive effort in prosthesis users that could be explored in future work.

## Conclusion

The literature revealed a remarkably characteristic visuomotor behaviour of upper limb prosthesis users across research studies. In contrast to the visuomotor behaviour of individuals with intact arm function, prosthesis users fixate more towards their hand and less towards target objects or locations. The reliance on vision to monitor the prosthetic hand prevents users from looking ahead towards future targets to plan for subsequent actions. Despite visuomotor behaviours that were mainly consistent, considerations should be made regarding the type of prosthesis and experimental task, as these may challenge the visuomotor system differently. Early work could not demonstrate that visuomotor behaviour was related to skill level or everyday usage. Therefore, it is unknown whether greater functionality is also marked by improved gaze behaviour and future work should investigate this gap in our knowledge.

Evidence has shown that gaze behaviour is related to prosthetic control and can be modulated with interventions, such as sensory feedback and training protocols. Importantly, eye tracking is a tool that provides a quantitative means of assessing human visuomotor behaviour and facilitates the understanding of the impact of prosthetic interventions to alleviate visual and cognitive demands. Research should thus consider including eye tracking as an outcome measure when evaluating novel interventions. Overall, the findings are promising, although more studies are needed with larger sample sizes to substantiate the repeatability and validity of the current findings. Eye metrics have also been used to study the cognitive load and sense of agency of upper limb prosthesis users. The literature to date suggests promising results in quantifying these phenomena, however more work is needed to validate the use of these eye metrics in an upper limb prosthesis user population.

## Data Availability

All data generated or analysed during this study are included in this published article and its supplementary information files.

## Abbreviations

ADL: Activities of daily living
AOI: Area of interest
EMG: Electromyography
SHAP: Southampton Hand Assessment Procedure
TLS: Target locking strategy
